# Identifying the challenges in implementing open science [version 1; peer review: 2 approved]

**DOI:** 10.12688/mniopenres.12805.1

**Published:** 2018-10-12

**Authors:** Sarah E Ali-Khan, Antoine Jean, E. Richard Gold

**Affiliations:** 1 Faculty of Law, Centre for Intellectual Property Policy (CIPP), McGill University, Montreal, Quebec, H3A 1W9, Canada; 2 Tanenbaum Open Science Institute (TOSI), Montreal Neurological Institute and Hospital, McGill University, Montreal, Quebec, H3A 2B4, Canada; 3 Department of Human Genetics, McGill University, Montreal, Quebec, H3A 1B1, Canada

**Keywords:** Open science, open access, intellectual property, innovation, collaboration, policy, commercialization, research process

## Abstract

Areas of open science (OS) policy and practice are already relatively well-advanced in several countries and sectors through the initiatives of some governments, funders, philanthropy, researchers and the community. Nevertheless, the current research and innovation system, including in the focus of this report, the life sciences, remains weighted against OS. In October 2017, thought-leaders from across the world gathered at an Open Science Leadership Forum in the Washington DC office of the Bill and Melinda Gates Foundation to share their views on what successful OS looks like. We focused on OS partnerships as this is an emerging model that aims to accelerate science and innovation. These outcomes are captured in a first meeting report: Defining Success in Open Science.

On several occasions, these conversations turned to the challenges that must be addressed and new policies required to effectively and sustainably advance OS practice. Thereupon, in this report, we describe the concerns raised and what is needed to address them supplemented by our review of the literature, and suggest the stakeholder groups that may be best placed to begin to take action. It emerges that to be successful, OS will require the active engagement of all stakeholders: while the research community must develop research questions, identify partners and networks, policy communities need to create an environment that is supportive of experimentation by removing barriers. This report aims to contribute to ongoing discussions about OS and its implementation. It is also part of a step-wise process to develop and mobilize a toolkit of quantitative and qualitative indicators to assist global stakeholders in implementing high value OS collaborations. Currently in co-development through an open and international process, this set of measures will allow the generation of needed evidence on the influence of OS partnerships on research, innovation, and critical social and economic goals.

## Foreword

In October 2017, thought-leaders from across the world gathered at an Open Science Leadership Forum in the Washington DC office of the Bill and Melinda Gates Foundation to discuss what successful open science (OS) partnerships would look like from their various vantage points. We focused on partnerships – in which all participants agree to work together along OS principles to acheive mutually-agreed upon goals, putting the product of their work in the public domain – as this is an emerging model that aims to accelerate science and innovation (Gold, 2016). Delegates from developed and developing nations, national governments, science agencies and funding bodies, philanthropy, the researcher community, patient organizations and the biotechnology, pharma and artificial intelligence (AI) industries identified the specific outcomes across social, economic, scientific and health spheres that would convince their organizations to invest in OS going forward. These discussions and outcomes are captured in a first meeting report: Defining Success in Open Science (Ali-Khan *et al*., 2018). In addition, delegates’ conversation turned to the challenges that must be addressed and new policies required to effectively and sustainably advance OS practice. We summarize these latter considerations in this second report, seeking to lay-out a roadmap to guide stakeholder activities to develop policy, resources and practice in this area. Once again, we extend our sincere thanks to everyone who attended the Leadership Forum for their enthusiasm and contributions (full list of antendees in Supplementary File 1).

## Context

The Leadership Forum was the first part of a step-wise process to develop and mobilize tools, best practices and other knowledge resources to assist global stakeholders in implementing high value OS collaborations, and build a global network of collaborators to advance this goal. A first key output of this work is a set of measures to construct a shared data resource upon which stakeholders can learn how OS collaborations contribute to innovation and advance discovery and public welfare goals. Currently in co-development through an open and international process, this set of measures will allow the generation of much needed evidence on the influence of OS partnerships on research, innovation and critical social and economic goals (Ali-Khan *et al*., 2018). In this report, we describe key challenges to the implementation of OS collaborations raised by delegates that need to be addressed to attain those goals. We summarize the discussions while noting that not every delegate agreed to every point or issue raised. As with the first report from this workshop, our goal is not to represent a consensus, but rather to capture the range of issues that delegates cited as important.

This work was inspired by the 2016 adoption of an institutionwide OS framework at the Montreal Neurological Institute and its associated Tanenbaum Open Science Institute (MNI/TOSI) and by the ground-breaking work of the Structural Genomics Consortium (SGC), launched in 2004. Given this starting point, our project focuses on the life sciences, and on industries or disciplines that would benefit from access to these data. We anticipate that in coming years, our or other groups may extend this work to other scientific domains and settings.

Both reports from the Leadership Forum, as well as development of the OS collaboration measures and associated resources are funded and supported by partners with a shared interest in advancing OS that are described in Supplementary File 2: the Bill and Melinda Gates Foundation, the Wellcome Trust, UK Research and Innovation, the Centre for Intellectual Property Policy, and TOSI.

## Introduction

### Defining open science

While some commentators reject the need for a precise definition of OS (Fecher & Friesike, 2014), unclear or overbroad definitions may make understanding its impact difficult (Patten *et al*., 2008) or limit its adoption (Ali-Khan *et al*., 2017). OS consists of the notion that scientific ideas, outputs, information, reagents, tools, bio-samples and other resources ought to be readily available for others to access, reuse and distribute without undue limitations. As noted, we focus on OS partnerships in which all members of the partnership agree to abide by OS principles within the scope of a mutually-agreed upon set of work. OS is facilitated through open access and open data: making publications and data freely available. In addition, to ensure the easy flow of information and knowledge created among partners in an OS collaboration, the two leading OS projects, the Structural Genomics Consortium and the Montreal Neurological Institute, have eschewed restrictive intellectual property rights over any co-created knowledge, materials or information (Dolgin, 2014; Edwards *et al*., 2009; Poupon *et al*., 2017).

Given our focus on collaborations and a literature search, we developed a working definition of OS as follows:

Open science (OS) comprises a set of institutional policies, infrastructure and relationships related to open access publication, open data and scientific resources, and lack of restrictive intellectual and other proprietary rights with the goal of increasing the quality and credibility of scientific outputs, increasing efficiency, and spurring both discovery and innovation.

This definition additionally accounts for the fact that, in our observations, most OS partnerships are led by public institutions and supported by significant public and philanthropic investment, appropriate institutional policies, and technological infrastructures that facilitate data and material sharing.

### Advancing open science

Areas of OS policy and practice, particularly open access and open data, are already relatively well-advanced in several countries and sectors through the initiatives of some governments, funders, philanthropy, researchers and the community – representatives of which were present at the Leadership Forum (See the Bill & Melinda Gates Foundation Open Access Policy, the Gates Open Research, the RCUK Common Principles on Data Policy, the RCUK Policy on Open Access and Supporting Guidance, the Wellcome Trust – Open research) (Butler, 2017; Dai *et al*., 2018). Nevertheless, delegates emphasized that the current research and innovation system, including in the life sciences, remains weighted against OS – business models, research culture and academic research incentives are generally ill-suited to support or capitalize on OS practices. They noted that for OS to achieve the successful outcomes envisaged (Ali-Khan *et al*., 2018), institutions must implement open behaviors broadly throughout the research and innovation lifecycle.

In the remainder of this report, we describe the challenges that delegates identified on the way to realizing the broad transformation of scientific culture that OS partnerships represent. In each section, we describe the concerns raised and what is needed to address them as discussed by delegates, supplemented by our review of the literature, and suggest the stakeholder groups that may be best placed to begin to take action.

## Changing research practice toward open science

Most delegates emphasized the importance of encouraging researchers – the primary agents of change in adopting OS practice as they make the decisions about what, when and how to share – and public research institutions to experiment with and eventually adopt OS practices. Researcher uncertainty and institutional inertia present some of the most significant barriers to adopting OS practice.

### Uncertainty and fear

Several delegates said that a significant proportion of researchers and their institutions are not well-versed in what it means to engage in OS practice, nor how best and at which point in the research cycle to participate. Delegates noted that researchers at their institutions are unsure at what point to release their data, and that lacking a clear understanding of the benefits, risks, boundaries and expectations of them regarding OS practice, many are wary about taking part. Several delegates reported the concern of some researchers and institutions that adherence to OS principles, including open data sharing and forgoing intellectual property protections on research outputs, may damage their relationships with important research partners such as industry collaborators or patients. In contrast, delegates from the MNI/TOSI reported that, on the contrary, industry collaborators have rapidly moved to establish OS-based partnerships with MNI researchers (Ali-Khan *et al*., 2017), and that patients are strongly supportive of OS, as indicated by high consent rates to inclusion of their data and materials in the MNI’s open data repository and biobank (Rouleau, 2017). Public research organizations build entire bureaucratic units that construct walls around knowledge through proprietary material transfer agreements, sponsored research agreements, and patenting and licensing practices. Under OS principles, these bureaucracies would need to overcome their inertia and adapt to facilitate knowledge exchange, adopt standard-form agreements and encourage sharing (See the Lambert Toolkit).

### Changing expectations around data and ownership

Many delegates stated that a widespread shift toward openness will require a change in the way that stakeholders think about their role in the research process and ownership of its inputs and outputs. Given that many researchers believe that if ‘you share you lose’, delegates underlined the need for a change in expectations about data-management and ownership. Thus, projects should be organized from the get-go with the expectation that data will be shared. Several funder delegates said that they are actively working to shift grantees’ expectations about data management and ownership. Many delegates called for an attitudinal shift in favour of sharing and collaboration: individual researchers should view their outputs as part of an effort to build a platform for discovery for the benefit for all, rather than considering that data belong to them (Edwards *et al*., 2017; Moulton, 2017). However, there is still the need for researchers to obtain benefits from their data and outputs through other means, such as recognition or greater research opportunities. Delegates further noted that researchers need to better recognize the value of sharing negative data: these can help others avoid duplication and research dead-ends with far-reaching implications for research cost and efficiency.

### Providing incentives for OS practice that leads to value

Many delegates advocated for ‘carrots rather than sticks’ to encourage researchers to adopt OS practices. This was the policy chosen by the MNI/TOSI in adopting OS throughout their labs. Policy should target open behaviours that lead to value – such as the development of data, drugs, health interventions, community practices – rather than imposing a general policy of openness for its own sake. While the study of OS is in its infancy (Ali-Khan *et al*., 2018), early research exist that can be used to identify points of value (Jones *et al*., 2014; Piwowar & Vision, 2013; Tripp & Grueber, 2011; Weiss, 2002; Williams, 2013).

### Making openness practical and beneficial

Delegates emphasized that it is not sufficient to convince researchers of the advantages of OS: OS needs to be built into researchers’ routine workflow. While lack of awareness of OS, inertia and complacency play a role in limiting uptake, the most important reason that researchers in the highly competitive life sciences hesitate to undertake OS practice is taking away time from other valuable efforts and being disadvantaged in comparison with those who do not share (Ali-Khan *et al*., 2015; Fecher *et al*., 2015; LERU Research Data Working Group, 2013; Levin *et al*., 2016). More particularly, researcher concerns include the following: managing and preparing data for sharing draws time and resources away from research; publicly-shared data may be scooped before researchers have the opportunity to publish using it; OS behaviors are poorly recognized and rewarded by academic hiring, promotion and tenure committees, and funders’ granting process; paying directly (rather than institutionally through subscription or other means) open access and article processing fees (APCs).

Several delegates highlighted prototype tools that facilitate the incorporation of OS practice into the ordinary research routine. For example, open workflow, analysis and data storage platforms offered by the Centre for Open Science (COS) at the University of Virginia allow researchers to register and manage projects, securely store data, privately collaborate, and to choose when to make all or parts of their projects publicly accessible (UVAToday, 2013). Likewise, all SGC researchers use electronic lab notebooks (ELNs) that encourage good note taking, create a digital history, facilitate data compilation and reporting, and readily allow for public sharing at the researcher’s discretion (Edwards *et al*., 2018). These new tools have the potential to revolutionize scientific workflow, while advancing OS principles including efficiency, reproducibility, openness and collaboration.

### Recognizing and rewarding researchers’ open practice

Most delegates agreed that providing researchers with meaningful recognition and rewards for OS practice is the most powerful lever for achieving a large-scale shift in behavior. Thus, delegates generally agreed that re-calibrating academic credit and attribution systems to measure and value OS practice, and ensuring that relevant indicators are incorporated into academic hiring, promotion and tenure process, and into prize and funding evaluation is an urgent priority. Delegates noted that there is a need to expand the range of scientific outputs and activities that are recognized – including for example, datasets, negative data, methods and protocols, materials, dissemination platforms, software, analytical tools, use of ELNs, policy publications, OA publications, pre-prints, etc. – and ensuring that these are trackable and citable by digital object identifiers (DOIs) or other means. New or alternative metrics are needed to measure value, impact and quality in an OS context: for example, views, downloads and re-use rates, as well as the scope and diversity of users may be important measures of impact while the provision of metadata and adherence to the FAIR Data Principles may reflect quality etc. In particular, public funder delegates underlined the need to demonstrate local benefits, particularly economic gains, to secure government support for OS. Funders called for the rapid development and testing of new output measures that they can use to shape future funding calls and evaluation process.

Many delegates emphasized the importance of making the criteria and measures by which researchers are evaluated, as well as the data and algorithms underlying them, transparent and open, whether for hiring, promotion or granting process. Many also highlighted the need to either borrow or create new mechanisms and incentives for building and organizing research teams within an OS ecosystem. In particular, they noted the need to create rewarding career paths for key roles whose importance is growing, and which may not neatly fit within the current academic framework, such as librarians, data scientists, curators, data managers and stewards. In this context, the role of traditional academic support staff such as librarians and archivists also need to be revisited.

### Developing and introducing OS policy

Many delegates contemplated approaches to launching OS frameworks at their organizations. Several, including some public funders, noted a risk of push-back or nominal, rather than substantive adherence, if policy introduction is mishandled. Many delegates recommended that OS policy be developed from the bottom-up, involving substantial engagement of key stakeholders. The uneven state of community knowledge about OS is one reason justifying this approach. The MNI/TOSI provides an example where the institution undertook an 18-month community consultation before unanimously adopting an OS policy framework (Rouleau, 2017). Several delegates called for protocols and best practices to guide stakeholder engagement and OS policy development. Several noted that change would best come from the scientific community, with the support and partnership of government and funders, rather than bing imposed by funders and government.

### Next steps

While recognizing the need for a bottom-up process, delegates noted a central role for government funders and philanthropy in supporting researchers and institutional practices toward OS. Noting that institutions, such as universities, ‘follow the money’, many delegates suggested that funders support those advocating behavioral and cultural change by making concrete statements of principle on appropriate uses of indicators and by running OS funding pilots. Because adapting to an OS environment comes at a cost, funders are in a better position to bear the risks and tolerate some loses in order to gain positives outcomes in the longer term.

To advance the OS agenda, delegates suggested that public funders and philanthropies should consider the following activities:

1. Launch OS-focused grant calls and prizes, at least on an experimental basis. We note that the Wellcome Trust recently launched a grant call (Research Enrichment – Open Research) while the Welcome Trust, National Institutes of Health and the Howard Hughes Medical Institute offered an open science prize (The Open Science Prize);
2. Develop clear structures and evaluation criteria through which to assess OS funding applications and ensure that peer review committees are familiar with the use of those criteria;
3. Develop and implement reporting metrics from grants that assess the variety of ways that OS partnerships contribute to research, innovation and social welfare;
4. Set aside funds to specifically reward researchers, especially emerging researchers, who adopt OS practices. This can be achieved by providing equipment, paying for open access fees, providing start-up funds, and awarding other benefits;
5. Share best practices, criteria and metrics among funders both in the public and philanthropic sectors;
6. Engage researchers, public research organizations (PROs) and other stakeholders including the ultimate beneficiaries of OS, communities and the public to build awareness of OS; and
7. Fund the development of a substantive, clear and reliable evidence-base through which decision-makers across sectors can determine when, in which circumstances, and with which ends, OS advances research and innovation goals better than do other research models.

In addition, PROs or units within them (such as the SGC and the MNI) should advance OS practice by undertaking the following activities:

1. Develop clear tenure and advancement criteria that take into account the diverse ways in which OS practice contributes to research, innovation and social welfare;
2. Develop clear and transparent principles that set out institutional commitment to OS and best practices to implement those principles, such as those developed by the SGC and MNI/TOSI, in respect of OS (Ali-Khan *et al.*, 2015);
3. Keep track of the processes through which those organizations or units adopted OS principles;
4. Develop standard-form material transfer, sponsorship, partnership and other agreements adapted to OS; and
5. Openly share the above with other organizations and units contemplating the implementation of OS practices.

As peer support for OS practice is critical to its adoption, researchers already practicing OS ought to consider the following activities:

1. Communicating with policy-makers, funders, patients, and the community about the benefits of OS to research; and
2. Provide training to emerging researchers on the benefits of OS practices including networking, recognition, greater ease of creating partnerships, and so on.

## Data-sharing and management

Data sharing is one pillar of open practice. This requires a focus on the infrastructure to support sharing, best practices regarding metadata and having skilled data managers who ensure the quality and sustainability of databases.

### Infrastructure and E-infrastructure

As described in the previous section, research and innovation requires that researchers are willing to share data is a major challenge to implementing OS; ensuring that they are able to do so is another.

Infrastructure, including trusted web-based repositories and storage capacity are essential prerequisites for making data publicly accessible and useable (Das *et al.*, 2017). Many delegates underlined that grant-based support, being generally short-term and disparate, is unsuited to keeping these fundamental resources sustainable, properly regulated and consistently at high-quality. Many delegates pointed to public sector funders and philanthropy as best-placed to develop and maintain trusted repositories that are run under well-defined and standardized conditions, offering the Protein Data Bank (Berman *et al.*, 2000) and the European Open Science Cloud as cases in point. Indeed, several funder delegates outlined the intention of their organizations to ensure that all grantees have the capacity to comply with their OS policy. For example, the Bill and Melinda Gates Foundation is developing a central data hub where all funded project data will be publicly-available. They are also supporting their open access policy by investing in APCs for grantees. Likewise, the Canadian Social Sciences and Humanities Research Council is partnering with industry to develop new data storage, sharing and analysis solutions. Several delegates noted that ensuring the long-term sustainability of repositories is challenge: new thinking around economic models is needed.

Cyber or E-infrastructures, as well as super-computing and distributed computing networks, allow data that is held in repositories to be linked, exchanged and analyzed. Many delegates emphasized that cyberinfrastructure and repositories must be compliant with appropriate ethical frameworks and include robust security and verification mechanisms to allow researchers to readily participate in OS. For example, they pointed to a need to establish secure mechanisms for data storage, including confidential or sensitive material such as that derived from human research subjects.

Delegates emphasized that overly complex data access mechanisms slow and increase the cost of research. They highlighted a critical need for clear and streamlined access mechanisms that will obviate the need for institutional signatures and lengthy paperwork, for example web-based click-through agreements. Likewise, simplified templates for OS-enabling material transfer and collaboration agreements, and other research-related documents are needed.

### OS principles and stewardship

Proper data sharing, management and stewardship are crucial to support OS practice. Many delegates noted that high-value data sharing should adhere to the FAIR Principles (Findable, Accessible, Interoperable and Re-useable) (Wilkinson *et al.*, 2016) and to the Transparency and Openness Promotion (TOP) guidelines (Nosek *et al.*, 2015).

Many delegates called for expanded roles for ‘data stewards’ who will optimize the curation of data, marketing and promoting that data to users, assisting researchers to find the best datasets for their purposes. In this context, several delegates pointed to a growing role for libraries and librarians.

### Critical importance of metadata

Several delegates, particularly from the AI community, underlined the critical importance of providing detailed metadata including the following: production dates, research questions, and details of the methods, reagents, protocols, workflows and platforms, instruments etc. used to generate data. This information allows users to place data in context, minimizing the potential for biased or incorrect follow-on research. In addition, delegates anticipate that versioning, annotation of data by users and about whom, how and when data were re-used etc. will increase the value of data over time. Data and metadata, including any licensing terms, should be machine-readable so as to enable electronic searches, deep learning and other electronic tools to gather and analyze data sets (Wilkinson *et al.*, 2016).

### Next steps

Delegates underscored the urgent need to establish norms, standards and policy to support OS data management. Among the actions that funders and philanthropy can undertake to support their development are the following:

1. Fund and manage the creation and maintenance of a shared OS data infrastructure, including tools and supporting research documents, including in developing countries;
2. Develop, at an international level, standards (perhaps by field or across domains) with respect to metadata and ensure that datasets are machine-readable;
3. Fund research on key aspects of data-sharing including the types of data most significant to different stakeholders and other attributes needed to maximize usefulness and interoperability of the data;
4. Develop mechanisms through which researchers can easily and without going through their central administrations, access data while respecting the privacy and consent; and
5. Spearhead policy and standard-setting around data management and sharing to ensure that datasets are maximally findable, accessible, interoperable, and re-useable over the long-term.

Delegates suggested that public research organizations, firms, and database managers engage more librarians, archivists, data scientists and other staff to support researchers in deciding which data to store in which formats and how to best access data.

## Consent, access and benefit sharing

Delegates noted that beyond data sharing, it is critical that OS practice ensures that the shared knowledge produced by research is usable and useful, and that access is ready, equitable and sustainable. These are all fundamental aspects of implementing high-value OS.

### Consent, control and privacy

Obtaining OS-compatible consent for the use and sharing of personal data and materials is absolutely critical to being able to grant access to data, consistent with the duties and rights owed to research participants. Open international data-sharing presents a number of challenges to traditional notions of consent (Kaye, 2012). Likewise, how to manage the sharing of older datasets collected using consent forms that did not anticipate OS-sharing is a key concern. Delegates from the MNI/TOSI described a year spent in collaboration with their institutional ethics committee to fine-tune the ethical and governance framework, patient consent forms and processes for their open biobank, the Clinical, Biological, Imaging and Genetic Data Repository (C-BIGR). They reported great enthusiasm among patients for participating in OS: they attained a greater than 90% consent rate from patients and families. Other delegates noted that ongoing research is needed to understand how this success can be replicated in other settings.

Some delegates called for engagement of research participant communities to inform data management and privacy policy. Others advocated for a shift in control over how and by whom data and samples are used, from the researcher to individual research participants. Web-based consent and engagement tools could facilitate this change (Kaye, 2012). Several delegates highlighted the need to ensure that indigenous, aboriginal and other marginalized communities are engaged in discussions concerning OS and consent. Many of these populations have experienced violations of consent and control of their samples and data in the context of biomedical research (The First Nations Information Governance Centre, 2014). Likewise, the cultural beliefs of some groups require the return of biological samples after research is complete (Canadian Institutes of Health Research *et al*., 2014), which may preclude the materials being shared.

### Equitable access, absorptive capacity and communication

Delegates identified the need for more substantive and equitable participation of a greater diversity of people in the research and innovation process, and greater engagement with science more generally. Equitable and ready access to data in forms that are understandable to the range of stakeholders is crucial to achieving these outcomes.

Delegates underlined the need to build equity in the capacity to access and make optimal use of shared data across stakeholder groups. Several delegates noted the challenges in lower resource settings. They cited deficits in broadband internet access, research infrastructure, and capacity more generally in many developing countries. Without these foundational resources, delegates pointed out that researchers may not be able to comply with OS policy even if this were in place. Thus, many delegates called for sustainable and long-term support to build and maintain OS research infrastructure in lower-income settings, coupled with funding of locally-led initiatives to strengthen research capacity.

Delegates highlighted other instances in which there is a need for support to build absorptive capacity. Several noted that open data is likely to be of great benefit, particularly to small and medium enterprises (SMEs). Yet, many firms lack the knowledge and skills to make use of this data and will need to develop new capacities to thrive in an OS environment. Delegates from Sage BioNetworks noted that there is a need to build researcher awareness and to develop skills to use available tools and technical platforms for OS. More generally, delegates called for the provision of shared data in audience-specific forms to maximize uptake. Delegates underlined that the reporting of scientific findings to lay populations should be accompanied by careful explanation of their context and implications to maximize benefit, while minimizing the potential for misunderstanding and confusion.

### Next steps

Funders and philanthropies play a critical role in ensuring compliance with ethical norms and the attainment of equitable access and sharing of benefits. Among the actions that these funders ought to consider are the following:

1. Fund research to explore research participants’ expectations and concerns about OS research, consent, and control, access and benefit-sharing; and explore new mechanisms to allow for OS sharing consistent with their needs and values;
2. Support the creation of templates, guidelines and other resources to assist stakeholders in implementing OS, for example templates for consent forms, material transfer, data access and collaboration agreements; case studies; ethics and governance frameworks; and protocols for sharing and communication of research results to participant communities, the media and the public;
3. Fund, develop and share good practices to ensure that patient organizations, community groups and local firms have or build the capacity to absorb and make optimal use of OS knowledge; and
4. Partner with PROs to provide (and require) training for researchers on good data practices including organization, management and sharing.

For their part, PROs ought to share templates, guidelines and other resources concerning consent with other organizations.

## Promoting diversity, equity and inclusiveness in the research process

While not unique to OS, many delegates suggested that OS practice be built with the goal of achieving greater and more substantive inclusion of a broader diversity of people in the research and innovation process, including early career researchers (ECRs), women, minorities, marginalized groups including indigenous and aboriginal populations, and researchers and populations from lower income settings. The delegate from the Genetic Alliance specified that ‘patient organizations expect OS to result in greater involvement of end-users and communities in the research process – for example, leading studies, framing research questions, making funding decisions and determining the outputs of value’. Equally, improved communication of research findings to participant communities and to the public is needed. This would not only maximize the potential benefits of research outcomes, but also respect the fundamental enabling contributions of participants and the public in donating their samples and data, and funding public research respectively.

Delegates spoke at length about the possibility that OS might lessen existing biases in the research ecosystem. For example, they pointed to granting and publication review processes that may favour male and established researchers (Helmer *et al*., 2017; Lariviere *et al*., 2013; Shen, 2013; Viner *et al*., 2004; “Women in neuroscience: a numbers game,” 2006). Likewise, they noted flawed research impact metrics such as the H-index (Hicks *et al*., 2015), which can promulgate inequities in career advancement and funding success. Some delegates called for external independent reviewing groups that are transparent about how granting and publication decisions are made and include the perspectives of a range of stakeholders including the public, patients and end-users to avoid reproducing the status quo.

### Next steps

Delegates called on all stakeholders to undertake the following actions in in their funding decisions, construction of OS partnerships, and pubic engagement:

1. Encourage practices that increase the diversity and transparency of the research process such as:
  - Involving the public and end-users in the research process from inception, for example: setting research priorities, reviewing grant applications and requiring they are offered meaningful roles within research teams;
  - Requiring that collaborators in developing countries or lower income settings occupy leadership roles in the research process, as jointly determined by both groups of collaborators, the community characteristics and the nature of the research; and
  - Encourage the consortia or public-private partnership research models whose aim is building knowledge and capacity within research communities while creating knowledge;
2. Develop research and capacity building with respect to OS partnerships with governments and NGOs in developing countries;
3. Incorporate, as in other grants, equity, diversity and inclusion requirements for research teams, and ensure that these are met;
4. Fund the development of protocols for public and research community engagement, and participation in the research and innovation process; and
5. Provide specific funding to support public and community engagement, return of results and benefit-sharing with research communities; and require that research teams provide plans for these activities in grants applications, and that they follow through on them.

In addition, delegates suggested that governments and inter-governmental organizations do the following:

1. Require the inclusion of end-user perspectives in regulatory approval processes, for example including patient outcome reports in drug approval processes (Hoos *et al.*, 2015); and
2. Develop a global standard research funding protocol and practices that encourages OS approaches that are broadly consistent between funders.

## Commercialization

To reap the social and economic benefits of OS research, we need to ensure that stakeholders have effective and efficient ways of harnessing outputs, and transferring them to those who can benefit from them, including but not limited to industry.

### Intellectual property rights

Some delegates saw intellectual property rights (IPRs) as a key challenge in the context of OS: many organizations and researchers that are interested in OS readily accept the notions of open access and open data, yet some may be reluctant to forego IPRs over jointly-created research. Some delegates pointed to the literature that IPRs claimed on academic research outputs present significant barriers to innovation. Several delegates underlined evidence generated over the last 30 some years indicating that financial returns to universities may just compensate for the out-of-pocket costs of patenting (Love, 2014; Nag, 2017), let alone loss of time and indirect costs.

OS collaborations such as those of the MNI/TOSI and the SGC adopted principles against the use of restrictive intellectual property rights over their research outputs, including those co-developed with industry, with the goal of easing the flow of information, knowledge and materials among partners (Dolgin, 2014; Poupon *et al*., 2017). Delegates noted, however, that the main barrier to adoption of the policies against restrictive intellectual property was not always industry: often, it is university central administrations, too many of which adhere to an outdated ‘linear’ model of academic patenting and licensing (Nicol, 2008). Some researchers, particularly those in clinical research, also express fear that giving up restrictive intellectual property rights would undermine their ability to conduct research jointly with industry. Most of the concerns are misplaced as the examples of the MNI and SGC illustrate, the latter with substantial industry investment in its first ten years (Jones *et al*., 2014).

### New financing and business models for innovation

Delegates strongly underlined the need for new financing and business models to ensure that promising outputs of OS are translated to public benefit. Some pointed to the limitations of venture capital financing for new biotech companies, including those stemming from OS research: funding cycles tend to be too short to advance candidates to another successful funding round. Investors expect an exit strategy and returns too quickly. Many delegates favoured augmented engagement between funders, investors and communities to focus and streamline the research and innovation process. Many delegates also called for strategies that favour development of products and services of real value to communities, for example that address unmet population needs.

Until recently, open approaches have attended only to upstream research and discovery phases of the innovation process (Masum & Harris, 2011) (See also the Allen Institute, the British Columbia Cancer Agency, the British Geological Survey, the Interdisciplinary Nanoscience Center at Aarhus University, the MNI, the Open Source Drug Discovery – India, the Open Source Malaria, Sage Bionetworks and the SGC). More recently, initiatives are exploring open commercialization. M4K Pharma (M4K), a spinoff from the SGC, aims to develop new affordable treatments for rare paediatric disease. Committed to efficiency and rapid advance of the field, M4K will not patent outputs. Instead, it will share all findings, including new chemical entities, and clinical and pre-clinical data, in the public domain. It holds regular quarterly meetings in public. Rather than acquire patents to ensure exclusive marketing rights, M4K will seek exclusivity over the use of its data package for regulatory approval, such as from the Food and Drug Administration, while rendering all data public and imposing affordable pricing on the manufacturer.

### Next steps

Delegates identified the following roles for funders and philanthropy:

1. Fund development of a robust prospective evidence base and case studies on the social and economic influence of open versus proprietary approaches to knowledge management and promote integrated translation of this knowledge to stakeholders, including governments, funders, philanthropy, researchers and PROs, firms and end-user organizations (Graham *et al.*, 2006); and
2. Support experimentation with new business models that seek to maximize efficiency, justice, affordability and end-user value.

Governments ought to consider creating policy across a diverse set of domains including taxation, direct subsidies, streamlined immigration processes, the abilityof charities to invest in innovative endeavours, etc. to encourage the development of local innovation hubs around OS research centers.

## Conclusion

Delegates at the Washington Leadership Forum recognized that while OS offers tantalizing benefits such as lower costs, increased productivity, better connection with communities and increased trust in science, it also requires a thoughtful and international policy foundation to thrive. In this report we canvassed some of the major issues that delegates thought critical to not only experimenting with OS collaborations, but making them a success.

While the research community carries the major responsibility for developing research questions, identifying partners and networking, policy communities need to create an environment that is supportive of experimentation. This involves identifying and removing barriers – including overly restrictive intellectual property practices, misinformation, and out-of-date peer review and promotion standards – to positively supporting OS collaborations through infrastructure investments, standardization of data and metadata requirements, consent and data protection rules, and OS funding calls.

To be successful, OS will require the active engagement of all stakeholders. This report is meant to contribute to ongoing discussions about OS and its implementation.

## Data availability

No data are associated with this article.

## Supplementary material

Supplementary File 1: Leadership Forum participant list

Supplementary File 2: Sponsor organizations list
