# Supplementary material for: Identifying the challenges in implementing open science [version 1; peer review: 2 approved]
Source: MNI Open Res. 2018 Oct 12;2:5. doi: 10.12688/mniopenres.12805.1 (PMC7845503; doi:10.12688/mniopenres.12805.1)
Supplement: Supplementary file 1 [file MNIOR-02-05-s001.pdf]

Supplementary File 1

LEADERSHIP FORUM PARTICIPANT LIST

Dr. Sarah Ali-Khan, Research Associate, Faculty of Law, McGill University

Dr. Lluis Ballell-Pages, Director, External Opportunities, GlaxoSmithKline, Tres Cantos Open Lab for Diseases of the Developing World

Dr. Patricia Brennan, Director, National Library of Medicine, Interim Associate Director for Data Science, National Institutes of Health, US Department of Health and Human Services

Dr. Katja Brose, Science Program Officer, Chan Zuckerberg Science Initiative

Ms. Rachel Bruce, Head of Open Science, Department for Business, Energy & Industrial Strategy, UK Government

Mr. David Carr, Program Manager - Open Research, Wellcome Trust

Dr. Simon Chaplin, Director of Culture and Society, Wellcome Trust

Me Mylène Deschênes, Ethics and Legal Advisor to Chief Scientist of Quebec, Fonds de recherche du Québec

Dr. Aled Edwards, Chief Executive Officer, Structural Genomics Consortium

Ms. Ashley Farley, Associate Officer of Knowledge & Research Services, Bill & Melinda Gates Foundation

Dr. Richard Gold, James McGill Professor, Faculty of Law, McGill University

Ms. Jennifer Hansen, Senior Officer, Knowledge & Research, Bill & Melinda Gates Foundation

Dr. Jason Karamchandani, Associate Professor, Department of Pathology McGill University

Dr. Michael Hawrylycz, Investigator, Allen Institute for Brain Science

Dr. Nadia Khelef, Senior Advisor for Global Affairs, Institut Pasteur

Mr. Robert J Kiley, Head of Open Research Development, Wellcome Trust

Ms. Elizabeth Kittrie, Strategic Advisor for Data and Open Science, US National Institutes of Health

Mr. Manoj Kumar, Head of Entrepreneurship and Innovations, Tata Trusts

Dr. Alexandre Le Bouthillier, Founder and COO, Imagia

Dr. Matthew Lucas, Executive Director, Corporate Strategy and Performance, Social Sciences and Humanities Research Council of Canada

Dr. Thomas Maina Kariuki, Director of the Alliance for Accelerating Excellence in Science in Africa

Dr. Lara Mangravite, President, Sage Bionetworks

Ms. Jessica Mankowski, Manager, Knowledge Translation Strategies, Canadian Institutes of Health Research

Dr. Sanjay Mehendale, Additional Director General, Indian Council of Medical Research

Dr. Mark Namchuk, SVP Research, Non-Clinical and Pharmaceutical Development, Alkermes

Ms. Thea Norman, Senior Program Officer, Bill & Melinda Gates Foundation

Mr. James O'Leary, Chief Innovation Officer, Genetic Alliance

Dr. Sébastien Paquet, Lead Applied Research Scientist & Culture Hacker, Element AI

Mr. Ben Pierson, Senior Program Officer, Bill & Melinda Gates Foundation

Dr. Claude Pirmez, Senior Researcher, Oswald Cruz Institute

Ms. Casey Selwyn, Fellow, Global Health Program**,** Bill & Melinda Gates Foundation

Ms. Annabel Seyller, Chief Operating Officer of the Open Science Experiment, The Montreal Neurological Institute

Dr. Carthage Smith, Senior Policy Analyst, Organization for Economic Co-Operation and Development (OECD)

Dr. Jeff Spies, Chief Technology Officer and Co-founder, Centre for Open Science

Dr. David Sweeney, Executive Chair Designate of Research England and Director, Research and Knowledge Exchange Higher Education Council for England

Dr. Michiel van Den Hauten, Head/Deputy Director of Research and Science Policy at the Ministry of Education, Culture and Science

Dr. Kate Williams, Scientific Director, Krembil Foundation
