# Supplementary material for: Identifying the challenges in implementing open science [version 1; peer review: 2 approved]
Source: MNI Open Res. 2018 Oct 12;2:5. doi: 10.12688/mniopenres.12805.1 (PMC7845503; doi:10.12688/mniopenres.12805.1)
Supplement: Supplementary file 2 [file MNIOR-02-05-s002.pdf]

Supplementary File 2

SPONSOR ORGANIZATIONS

Bill and Melinda Gates Foundation: Guided by the belief that every life has equal value, the Bill & Melinda Gates Foundation works to help all people lead healthy, productive lives. In developing countries, it focuses on improving people's health and giving them the chance to lift themselves out of hunger and extreme poverty. In the United States, it seeks to ensure that all people—especially those with the fewest resources—have access to the opportunities they need to succeed in school and life. The foundation believes open access to research accelerates its impact and means more lives saved and improved. Based in Seattle, Washington, the foundation is led by CEO Dr. Susan Desmond-Hellmann and Co-chair William H. Gates Sr., under the direction of Bill and Melinda Gates and Warren Buffett.

Centre for Intellectual Property Policy (CIPP): McGill University's CIPP brings together scholars, students and the community to independently advance understanding of the role and attributes of intellectual property and of innovation in economic and social development.

Tanenbaum Open Science Institute (TOSI): Created by McGill University, TOSI aims to develop and spread a new model of discovery and innovation based on the usage of OS principles as accelerators, for the benefits of patients and community, and the development of best practices globally.

UK's Department of Business, Energy and Industrial Strategy: The BEIS is a ministerial department supported by 45 agencies and public bodies, including the UK Research Councils, Research England and Innovate UK, that aims to build an economy that works for everyone, so that there are great places in every part of the UK for people to work and for businesses to invest, innovate and grow. BEIS, and its related research organizations, support Open Science policies as a way to contribute to meeting these goals.

The Wellcome Trust: Wellcome exists to improve health for everyone by helping great ideas to thrive. The Trust is a global charitable foundation, both politically and financially independent that supports scientists and researchers, takes on big problems, fuels imaginations, and sparks debate. Wellcome supports open research as we believe that transforming human health will take longer if research outputs – like publications, data, software and biological materials– aren't managed, shared and used in ways that unleash their full value.
