# Supplementary material for: Identifying the challenges in implementing open science [version 1; peer review: 2 approved]
Source: MNI Open Res. 2018 Oct 12;2:5. doi: 10.12688/mniopenres.12805.1 (PMC7845503; doi:10.12688/mniopenres.12805.1)
Supplement: Peer review history

# Current Peer Review Status:

**DOI:** 10.21956/mniopenres.13866.r26146

**Published:** 2019-02-04

**Authors:** René von Schomberg

**Affiliations:** European Commission, Brussels, Belgium

I believe that the authors did make a good effort to comprehensively map the challenges for stakeholders to implement open science. My remarks, pre-dominantly relate to issues which could have been phrased better or topics which could have been added.

1. Defining open science.
  I believe that the authors do (rightly so!) more in the open letter than the referenced definition of open science (page 3) allow them to do. According to my assessment, there are two elements not articulated in this definition. 1. The rationale of open science is to share knowledge ‘as early as possible’ 2. That the rationale of open science is to include al diverse set of knowledge producers, e.g. bringing different disciplines but also different knowledge actors (e.g industry, academia, citizens, non-governmental organisations, public authorities etc together to co-produce new knowledge.
  I am confident the authors actually agree on those, but these elements are missing in the definition. The ‘early as possible’ is well demonstrated in open science research on Ebola and other public health issues, as well as the necessity to use open science.(e.g save lives, in time, in the case of Ebola, conventional science would have come too late!). The authors rightly refer to SGC as a groundbreaking practice: also in this case, the ‘early as possible’ is relevant here. The ‘early as possible’ implies often prior to publication. This issue is not raised, but constitutes a major challenge as journals only publish ‘original’ research. OS scientist could therefore ‘lose’ opportunities to publish. However, OS ‘relativises’ the importance of publishing and makes ‘knowledge sharing’ a priority over publishing. OS is not simply about making publications and data, open and accessible, but changes the way we have to operate as scientists in terms of what are the priorities in the research process.
  The second element refers to the inclusiveness of other actors. Again, I feel confident that the authors share this opinion and they raise it further down the open letter. However, it fails to address fully the ‘why’ of the inclusiveness. I think it can better articulated that open science, includes other actors to make science not only more responsive to societal challenges but also engage with the other actors to make the science ‘better’, both in quality as well giving scientific research a direction, either to a scientific or to a societal challenge. It may be argued that open science is especially useful as a science mode for mission oriented science towards societal challenges, although open science should foster scientific excellence across the whole spectrum of science. This topic is also not (directly) addressed. This is of high importance when it comes to involving science funders to promote Open Science.
  Inclusiveness can be better described as open collaboration with all knowledge actors relevant for the issue to be investigated.
  Furthermore, the authors could very well articulate that open science not just relates to the openness of scientific outcomes but also the openness of scientific inputs. For example, the open data of the European Space Agency allows ecologist to study topics such as desertification to an extent the disciple was not able to conduct before such open data structures existed.
  The issue of citizen science, as ‘citizens doing science’ (so not simply public engagement with science) is not raised. I believe it is relevant both for inclusiveness and for quality of science.
2. Data sharing/Management
  I believe there is an omission: the authors do not mention that the use of Data Management Plans should become mandatory for scientist who receive public funding. It is an essential tool to advance open science on the ‘data’ front.
  The authors seems to equate open data infrastructures with an initiative such as the European Open Science Cloud. The latter which has been launched in November 2018 (only mentioned, but no full reference to it and it is operational rationale!) does more than any open science infrastructure did before: namely making it possible to deposit, share and re-use data across scientific disciplines and countries. It integrates a governance structure addressing data-protection, privacy and ethical rules. It can therefore become an ‘engine’ for open sciencebringing disciplines and knowledge actors from various countries together.
3. Role of Stakeholders
  I believe the authors can articulate (I am confident they would agree on this point) that all stakeholders need to become mutually responsive to each in order to advance and reward open science (e.g. publishers, universities, funders etc). It would be helpful what challenges are specific for particular stakeholders but also where they need to ‘coordinate’ on. Although this is a central element of the open letter, I did not find any reference to the Open Science Policy Platform that consist of 25 major stakeholder organization to do precisely this. This Platform was already launched in 2016 and delivered major advice towards their stakeholders on 8 aspects of open science. I think it is worth referring to, as the whole exercise of this open letter has, among other purposes, to advocate the engagement of stakeholders on open science in this way. So why not give an existing example (or otherwise, inform on what is wrong with the existing one).

**Is the rationale for the Open Letter provided in sufficient detail?**

Partly

**Does the article adequately reference differing views and opinions?**

Yes

**Are all factual statements correct, and are statements and arguments made adequately supported by citations?**

Partly

**Is the Open Letter written in accessible language?**

Yes

**Where applicable, are recommendations and next steps explained clearly for others to follow?**

Yes

***Competing Interests:*** No competing interests were disclosed.

***Reviewer Expertise:*** Your Area(s) of Research (optional) Please state your area(s) of expertise (e.g. epilepsy, fMRI or cellular neuroscience), particularly if you feel that you are able to assess only certain aspects of this article. Open Science, Responsible Research and Innovation, Technology Assessment, Ethics, Policy Analysis

**I have read this submission. I believe that I have an appropriate level of expertise to confirm that it is of an acceptable scientific standard.**

# Peer review

**DOI:** 10.21956/mniopenres.13866.r26141

**Published:** 2018-11-28

**Authors:** Roberto Caso, Giulia Dore

**Affiliations:** Faculty of Law, LawTech Group, University of Trento, Trento, Italy; University of Trento, Trento, Italy

We were asked to comment on the report “Identifying the challenges in implementing open science”.

The report accounts for the discussions emerged after the October meeting in 2017, when thought-leaders from across the world gathered at an Open Science Leadership Forum in the Washington DC office of the Bill and Melinda Gates Foundation (its results are summarized in a first meeting report^1^, suggesting some actions to implement in future policies that wish to provide a solution to the problems therein arose.

We did not participate to this Forum.

The authors adopt the following definition of Open Science (OS):

“Open science (OS) comprises a set of institutional policies, infrastructure and relationships related to open access publication, open data and scientific resources, and lack of restrictive intellectual and other proprietary rights with the goal of increasing the quality and credibility of scientific outputs, increasing efficiency, and spurring both discovery and innovation”.

In their conclusions, the authors refer that those who participated to the Forum believed that OS needs a thoughtful and international policy foundation to thrive.

New generation policies – the authors argue – should acknowledge the existence of some barriers to the fullest development of OS. These barriers, with reference to the research communities, include:

- Overly restrictive intellectual property practices;
- Misinformation;
- Out-of-date peer review and promotion standards.

The authors foresee the following implementation strategies:

1. Changing research practice toward open science;
2. Data-sharing and management;
3. Consent, access and benefit sharing;
4. Promoting diversity, equity and inclusiveness in the research process;
5. Commercialization.

These strategic actions are certainly valuable, but may not be sufficient, for OS to succeed.

On the matter of identifying the barriers to OS, it is worth doing some considerations to clarify certain aspects.

Among the factors that impede the advance of OS, we may identify the following:

- An evaluation system that treats science only as a competitive game among individuals or research groups. Instead, good research is based on the balance between competition and cooperation.
- Evaluation of research based on bibliometric data that confines valued research to the steady progression – in the short period – in rankings based on citation indexes. It follows the perverse effect that doing good research does not matter, what matters is reaching the highest ranking. See, e.g., M. Biagioli (2016)^2^.
- Evaluation and quality measurement of research based on proprietary database and anonymous peer-review. The logic of OS should apply also to evaluation. Closed (secret) bibliometric data and anonymous peer review nourish the concentration of evaluative power in the hands of few private (e.g. Clarivate Analytics o Elsevier) or public (such as some national evaluation agencies) entities.
- Decreased public funding to research that is free and independent, thus curiosity-driven.
- Increasingly restrictive intellectual property rights, with no exceptions for scientific research and academic teaching. See, e.g., Reichman *et al*. (2013)^3^.
- Competitive attitude of researchers within no-profit organizations like universities. Researchers – many of them precarious workers – are more and more tangled into competitive runs for funding and much less into pure research. Incentives that are typical of for-profit businesses – included the commercial exploitation of intellectual property rights – are increasingly widespread. There is a growing risk of conflict of interests and violations of scientific integrity. See, e.g., M.A. Edwards & S. Roy (2017)^4^.
- Internet is more and more governed by the interests of commercial platforms. If OS wishes to take back the balance between public and private research, between no-profit institutions and commercial entities, then the problem of concentration of power on the web must be addressed. Otherwise there is a dangerous likelihood that no-profit institutions produce open data that are subsequently exploited by big Internet commercial platforms. See, e.g., M. Hagner (2018)^5^.

Identifying a list of factors that better specify the barriers to the full development of OS helps to understand why certain actions may not be enough.

For instance:

- If a research community forsakes to use restrictive intellectual property rights (e.g. decide not to register patents) and publishes open data, this is certainly a contribution to the success of OA, but this cannot be enough if there lacks a system of copyright exceptions and limitations to allow for example data mining.
- Educating researchers to OS and offering the right incentives to publish in OA for the progression of their career is certainly a useful contribution to OS, but it is not enough to improve science if the rate of openness (in terms of the number of OA publications and open data) becomes simply another bibliometric indicator among others.

These thoughts perhaps may add new elements to the definition of Open Science.

Open Science should not only aim at:

- increasing the quality and credibility of scientific outputs,
- increasing efficiency,
- spurring both discovery and innovation.

It should also aim at safeguarding the democratic features of science and its Mertonian norms, promoting autonomy and scientific freedom of scientists.

Otherwise, there is a visible potential for recreating old oligarchic and oligopolistic mechanisms – although in the context of formal openness of publications, data and other scientific results – that can only endanger science.

**Is the rationale for the Open Letter provided in sufficient detail?**

Yes

**Does the article adequately reference differing views and opinions?**

Partly

**Are all factual statements correct, and are statements and arguments made adequately supported by citations?**

Yes

**Is the Open Letter written in accessible language?**

Yes

**Where applicable, are recommendations and next steps explained clearly for others to follow?**

Yes

***Competing Interests:*** Roberto Caso is associate member of McGill University, Faculty of Law, Centre for Intellectual Property Policy, https://www.cippmcgill.ca/team/. Roberto Caso and Giulia Dore were co-organizers of a conference in Italy (“Not for Profit: The Role of Universities in Open Science, University of Trento, 25 October 2018, https://webmagazine.unitn.it/en/evento/giurisprudenza/44286/not-for-profit-the-role-of-universities-in-open-science) where Prof. Richard Gold was an invited speaker.

**We have read this submission. We believe that we have an appropriate level of expertise to confirm that it is of an acceptable scientific standard.**

## References

[cit0044] Ali-KhanS, JeanA, MacDonaldE, GoldE: Defining Success in Open Science. *MNI Open Research*. 2018; 2.10.12688/mniopenres.12780.1PMC585263929553146

[cit0045] BiagioliM: Watch out for cheats in citation game. *Nature*. 2016; 535 (7611).10.1038/535201a27411599

[cit0046] ReichmanJH, OkedijiRL: When Copyright Law and Science Collide: Empowering Digitally Integrated Research Methods on a Global Scale. *Minn Law Rev*. 2012; 96 (4): 1362-1480.24639595PMC3955386

[cit0047] EdwardsMA, Roy S: Academic Research in the 21st Century: Maintaining Scientific Integrity in a Climate of Perverse Incentives and Hypercompetition. *Environ Eng Sci*. 2017; 34 (1): 56-61.10.1089/ees.2016.0223PMC520668528115824

[cit0048] HagnerM: Open Access, data capitalism and academic publishing. *Swiss Med Wkly*. 2018; 148: w14600.2945242610.4414/smw.2018.14600
